# A Bivalent Protein r-PAbxpB Comprising PA Domain IV and Exosporium Protein BxpB Confers Protection Against *B. anthracis* Spores and Toxin

**DOI:** 10.3389/fimmu.2019.00498

**Published:** 2019-03-19

**Authors:** Saugata Majumder, Shreya Das, Vikas Kumar Somani, Shivakiran S. Makam, Joseph J. Kingston, Rakesh Bhatnagar

**Affiliations:** ^1^Defence Food Research Laboratory, Microbiology Division, Defence Research Development Organisation, Mysore, India; ^2^School of Biotechnology, Jawaharlal Nehru University, New Delhi, India

**Keywords:** anthrax, BxpB, PA, spore challenge, protection

## Abstract

Anthrax vaccines primarily relying only on protective antigen (PA), the cell binding component in anthrax toxins provide incomplete protection when challenged with spores of virulent encapsulated *Bacillus anthracis* strains. Alternatively, formaldehyde inactivated spores (FIS) or recombinant spore components generate anti-spore immune responses that inhibit the early stages of infection and augment the PA protective efficacy. In the present study domain IV of PA was spliced with exosporium antigen BxpB via a flexible G4S linker to generate a single functional antigen r-PAbxpB that was further assessed for its protective efficacy against anthrax toxins and spore infection. Immunization of mice with r-PAbxpB elicited significantly high titer antibodies comprising IgG1:IgG2a isotypes in 1:1 ratio, balanced up-regulation of both Th1 (IL2, IL12, IFN-γ) and Th2 (IL4, IL5, IL10) cytokines and high frequencies of CD4+ and CD8+ T cell subsets. The anti-r-PAbxpB antibodies significantly enhanced spore phagocytosis, and killing within macrophages; inhibited their germination to vegetative cells and completely neutralized the anthrax toxins as evidenced by the 100% protection in passive transfer studies. Active immunization with r-PAbxpB provided 100 and 83.3% protection in mice I.P. challenged with 5 × LD_100_ LD of toxins and 5 × 10^4^ cfu/ml Ames spores, respectively while the sham immunized group succumbed to infection in 48 h. Therefore, the ability of r-PAbxpB to generate protective immune responses against both spores and toxin and provide significant protection suggests it as an efficient vaccine candidate against *B. anthracis* infection.

## Introduction

The Gram positive, spore forming bacilli *Bacillus anthracis* is the etiological agent of anthrax, a fatal zoonotic disease that primarily affects ungulates and humans and is categorized as Category A bio threat agent by Center for Disease Control and Prevention (1). The structurally complex spore that is stable to harsh environmental conditions establishes infection in the host via inhalation, ingestion, or cutaneous routes; the later accounting for majority of the human clinical cases globally (2). Within the host, the spores hijack the macrophages (3) that otherwise obliterate the invading pathogen, germinate to vegetative cells, and replicate while being transported to the lymphatics (4). The vegetative cells undergo further multiplication and express the AB type cytotoxins namely, the lethal and edema toxins (LT and ET) which systematically inhibits both humoral and cell mediated arms of the immune system, alters the cell signaling and helps to establish a protected niche for further multiplication and systemic spread (5–7).The systemic spread of infection results in septic shock and necrosis of spleen, liver, and bone marrow that ultimately leads to the death of the host due to multi organ failure (8, 9).

The protective antigen (PA) forms a common host cell binding moiety for both Lethal factor (LF) and Edema factor (EF) components of the anthrax toxin and aids in their transport to the host cell cytosol. The binding of this 83 kDa protein with the LF and EF happens through the domain IV (PAIV) while the other domains (I–III) facilitate proteolytic activation, heptamerization, and host cell binding (10). It is the key immunogen in anti-anthrax vaccines that are available commercially and provides protection predominantly through the production of toxin-neutralizing antibodies (11). The PA based vaccines though effective, does not provide complete protection against capsulated strains of *B. anthracis* (12) and provide incomplete protection when spores are used in challenge studies (13). Alternatively, anti-spore immune responses elicited by formaldehyde inactivated whole spores, or exosporium antigens augments the protective efficacy of PA based vaccines when mice or guinea pigs are challenged with virulent strains of *B. anthracis* spores (14, 15). The 17 kDa protein BxpB (also known as ExsFA) is located in the exosporium basal layer and is necessary for the residual attachment of BclA filaments to the exosporium thereby maintaining the exosporium assembly (16). This exosporium protein is not protective by itself, but augments the protective efficacy of PA based vaccines by enhancing spore uptake and killing by macrophages as evidenced by the increase in mean time to death against spore challenge (17).

The current trend in modern vaccinology aims to design rational vaccines based on the detailed understanding of pathogen biology and the mechanism leading to protection (18–20). To mediate comprehensive protection, anti-anthrax vaccines are required to elicit both cellular and humoral immune response that prevents the establishment of infection by spores and results in toxin neutralization (21, 22). Therefore, in the present study, the domain IV of PA (PAIV) was spliced to BxpB on a single protein scaffold to generate fusion protein r-PAbxpB following structural vaccinology rationale to have improved protective efficacy. The later approach of generating a fusion protein offers the advantage of obtaining a more defined product, reducing the number of recombinant expression and purification, and cost of production compared to mixing of multiple individual antigenic components (23). The fusion protein was further characterized for its correlates of protection and ability to provide concurrent protection against both *B. anthracis* spores and toxins.

## Materials and Methods

### Bacterial Strains and Their Growth Conditions

*Bacillus anthracis* strains BA10 and Ames harboring both pXO1 and pXO2 plasmids were used the present study. The strains were maintained in Brain Heart Infusion (BHI) broth and incubated at 37°C with shaking. *Escherichia coli* strains, BL21DE3, and DH5α used for cloning and maintaining the plasmids were maintained in (LB) Luria- Bertani broth. All the media were procured form Hi-Media, India. Antibiotics were procured from Sigma-Aldrich (Bangalore, India).

### Preparation of Spores and Toxins of *B. anthracis* BA10 (*pXO1^+^, pXO2^+^*)

The crude toxin from *B. anthracis* BA10 was extracted according to Makam et al. (24). Briefly, *B. anthracis* BA10 cells were grown overnight at 37°C in toxin production media (25) and centrifuged at 7,800 rpm for 10 min. The toxin present in supernatant was precipitated by saturated ammonium sulfate, dialyzed in 1X PBS (pH 7.0 ± 0.2) and filtered by passing through 0.22 um filters. All the above steps were performed in 4°C to avoid toxin degradation. The toxins were concentrated using lyophilization and stored in −20°C for further applications.

For harvesting spores, *B. anthracis* grown in Modified Germination (G) medium (26) for 48 h at 37°C was incubated at 65°C for 1 h to inactivate the vegetative cells and then washed with cold distilled water (4°C) to remove the inactive vegetative cells. Subsequently, the number of spores remaining in the suspension were quantified by plating on BHI agar and stored in −20°C for further use.

### Cell Lines and Media

Macrophage cells lines RAW 264.7 procured from National Center for Cell Science, Pune, India were grown in Dulbecco's Modified Eagle's medium (DMEM) with 10% FBS, 50 units/ml penicillin, and 50 μg/ml streptomycin. The cells were maintained at 37°C in 5% CO_2_. All the cell culture media, reagents and chemicals were procured from Sigma-Aldrich (India).

### Animals and Ethic Statement

Specific Pathogen Free (SPF), female, 4–6 week old BALB/c mice were procured from the Central Animal Facility, Defense Food Research Laboratory, (DFRL), Mysore, and maintained under sterile conditions. The mice were acclimatized to the laboratory conditions for 1 week before the initiation of experiments, provided with sterile food and water *ad libitum* and all possible efforts were imposed to minimize sufferings. All the animal experimental procedures performed in the present study were in accordance with the animal usage protocols approved by the Institutional Animal Ethical Committee, Defense Food Research Laboratory (DFRL/28/IAEC/CPCSEA), and Jawaharlal Nehru University (JNU) (19/1999/CPCSIA) completely accredited by Committee for the Purpose of Control and Supervision of Experiments on Animals (CPCSEA), India.

### Construction of *PAbxpB* Chimeric Gene

The gene sequences encoding PAIV and BxpB were retrieved from NCBI database and primers were custom synthesized from Sigma, Bangalore India. Chimeric gene *PAbxpB* was constructed by overlap extension PCR cloned in *E. coli*, BL21 DE3 expressed, and purified as soluble protein under denaturing conditions as mentioned in Supplementary File 1.The LPS content of the recombinant protein was determined by using the *Limulus* Amoebocyte Lysate (LAL) test (Lonza, India, Product # F245-06SA) according to manufacturer's protocol.

### Mice and Immunization Protocol

A group of mice (*n* = 12) was immunized sub-cutaneously (s.c.) with 30 μg r-PAbxpB antigen (in 0.1 ml volume) with Freund's complete adjuvant in 1:1 (v/v) ratio for evaluating the immunogenicity of r-PAbxpB by characterizing the antibody and cytokine responses, proliferation of splenocytes and relative expression of CD4+ and CD8+ cells. Consequently, the animals received boosters with similar concentration of antigen with Freund's incomplete adjuvant on 7, 21, and 35th day. The control group of mice (*n* = 12) was sham immunized with a similar volume of 1X PBS (pH 7.4 ± 0.2) in adjuvant. Blood samples were drawn periodically (days 0, 14, 28, 42) through retro-orbital plexus and the sera were collected and stored in −20°C for further use.

### Estimation of Antibody Titers and IgG Isotypes

The anti-r-PAbxpB specific antibody titers were measured using 2-fold serial dilutions of sera from six randomly selected r-PAbxpB immunized mice by indirect ELISA. Endpoint titers were determined as maximum antibody dilution when O.D. value was twice more than the mean O.D. value of the sham immunized mice sera. The anti-r-PAbxpB specific antibody isotypes IgG1, IgG2a, IgG2b, IgG3, and IgM were evaluated on serum samples (1:1,000th dilution) collected from r-PAbxpB and sham immunized mice on 42nd day of immunization by Mouse Antibody Isotyping kit (Sigma, India) as per manufacturer's protocol. Absorbance was measured thrice at a wavelength of 492 nm in 1 min time interval (Infinite M200 Pro; Tecan, Grodig, Austria) and the mean O.D. ± S.D. values were plotted in graph.

### Opsonophagocytic and Macrophage Killing Assay

The opsonophagocytic potential of anti-r-PAbxpB antibodies was evaluated on RAW 264.7 macrophage cells as per Welkos et al. (13) with minor modifications. Briefly, heat activated, refractile ungerminated *B. anthracis* BA10 spores (3 × 10^8^ spores/ml) were pre-incubated with 10-fold serial dilutions of r-PAbxpB immune sera or sham immune sera, for 30 min in 4°C and then added to RAW 264.7 macrophage cells (5 × 10^5^ cells/well) and incubated for 45 min at 37°C in 5% CO_2_. Post incubation the infected macrophage cells were washed with sterile 1X PBS (pH 7.0 ± 0.2) and incubated with DMEM containing 10% FBS and 10 μg/ml gentamicin at 37°C in 5% CO_2_ for 30 min to remove extracellular vegetative cells. Subsequently, the macrophage cells were washed with sterile ice cold 1X PBS and incubated for 5 min in 100 μ1 of 0.1% Triton X [Sigma, India] to lyse the macrophage and plated on LB agar for determining the viable spore count. Data is presented as % spore uptake by the macrophage cells.

In case of macrophage killing assay *B. anthracis* spores (3 × 10^8^ spores/ml) were pre-incubated with 1: 10 and 1:100 dilutions of r-PAbxpB immune sera or sham immune sera for 30 min in 4°C. The antibody treated spores were added to RAW 264.7 macrophage cells (5 × 10^5^ cells/well) and incubated for 0, 1, 4, and 18 h, respectively, at 37°C in 5% CO_2_. Post incubation, the macrophage cells were washed with sterile 1X PBS (pH 7.0 ± 0.2), treated with DMEM+ 10% FBS+ 10 μg/ml gentamicin at 37°C in 5% CO_2_ for 30 min, lysed with 0.1% Triton X (Sigma, India) and plated on LB agar to determine the percentage survival of spores (Survival %).

### Germination Inhibition Assay

To enumerate the ability of the anti-r-PAbxpB antibodies to inhibit the spore germination, heat activated ungerminated spores (3 × 10^8^ spores/ml) pre-treated with r-PAbxpB antibodies were incubated at 30°C for 25 min in germination media (1%(v/v) BHI in water in 96 well micro-titer wells. Spores treated with PBS and germination media are considered as positive control. At every 2.5 min interval absorbance was taken at 560 nm in micro-titer plate reader (Infinite M200 pro; Tecan, Grodig, Austria). The experiment was performed in triplicates and extent of germination inhibition was analyzed by the decline in O.D._560_ and plotted in a graph.

### Splenocytes Proliferation Assay

The splenocytes (5 × 10^5^ cells/ well) collected from the immunized and sham immunized mice (*n* = 6 each group) were harvested in DMEM media, stimulated with r-PAbxpB (5–40 μg/well) and incubated at 37°C for 72 h at 5% CO_2_. ConA at 20 μg /ml concentration was used as positive control. Splenocytes with DMEM media alone were considered as negative control. After 72 h of incubation the media was replaced by DMEM with 50 μg of MTT [3-(4, 5-dimethythiazol-2-yl)- 2,5-diphenyl tetrazolium bromide] in dark for 2 h at 37°C. The MTT reagent was replaced with 200 μl of Dimethyl Sulfoxide (DMSO) and O.D. was observed at 570 nm in micro-titer plate reader (Infinite M200 pro; Tecan, Grodig, Austria).

### Cytokine Level Measurement

Post immunization, the mice splenocytes were processed for the analysis of the Th1 cytokines (IL-2, IL-12, and IFN-γ) and Th2 cytokines (IL4, IL-5, and IL-10) elicited in r-PAbxpB and sham immunized groups. The cytokines were estimated using Bio-Plex Pro Mouse Cytokine 8-Plex Immunoassay kit (Bio-Rad, USA) as per manufacturer's instructions. Briefly, magnetic beads coated with anti-cytokine antibodies were added to the wells and incubated for 1 h at 37°C with equal volume of cell supernatant from r-PAbxpB and sham immunized mice splenocytes exposed to r-PAbxpB (30 μg/ml). Post incubation the microtiter plates were washed with Bio-Plex Wash Buffer and incubated with detection antibody for 1 h at 37°C. After consequent washing with Bio-Plex Wash Buffer the wells were incubated with Streptavidin-PE, in dark. After the wells were washed the beads in each well were resuspended in assay buffer for 30 s with shaking at 1,100 rpm. After incubation the fluorescence was measured on a Bio-Plex 200® array reader and analyzed with the Bio-Plex Manager software v3.0.

### Flow Cytometric Analysis of CD4+ and CD8+ T-cell Immune Response

To evaluate the CD4+ and CD8+ T cell counts in immunized mice, Flow Cytometry analysis was performed according to Williamson et al. (27). Briefly, peripheral blood samples were collected from r-PAbxpB and sham immunized mice (*n* = 3 each group) on the 45^th^ day of the immunization schedule and treated with 2 mM EDTA as an anti-coagulant. The erythrocytes were lysed using 1:10 diluted lysis solution (FACS lysing solution, BD) incubated at 37°C for 30 min in dark. Post incubation the cells were washed twice with Dulbecco's PBS and 10^6^ cells were stained with FITC anti-mouse CD4+ antibodies (BioLegend, India) and PE anti-mouse CD8+ antibodies (BioLegend, India). A minimum of 20,000 events were counted for each analysis using BD FACS Verse Flow Cytometer (Becton-Dickson, Singapore) and results were analyzed using Kaluza software version 3.1v (Beckman Coulter, USA). The percent activated CD4+ and CD8+ T cell populations were determined on scatter plot.

### Passive Transfer Studies

Anti-r- PAbxpB sera and sham sera were 10-fold diluted and incubated at 56°C for 30 min to inactivate the complement system and injected in i.p. (200 μl) in two groups of naïve female BALB/c mice (*n* = 6 each group). Post 24 h of passive transfer of anti-r-PAbxpB and sham sera each group was challenged with 5 × LD_100_ dose of crude anthrax toxin. Animals were closely monitored for signs of weakness and survival for 15 days. The toxin neutralization efficacy of anti-r-PAbxpB was calculated by Kaplan-Meier's method to compare percentage survival. The LD_100_ was defined as the smallest dilution of toxin in 0.2 ml volume causing 100% lethality in mice within 24 h post injection. In our study one LD_100_ was found equivalent to be 100 μg for each mice body weight.

### Active Protection Against *B. anthracis* Spores and Toxin

Two groups of female BALB/c mice were (*n* = 12) were immunized with 30 μg r-PAbxpB while two control groups (*n* = 12) were sham immunized with 1X sterile PBS (pH 7.4 ± 0.2) in adjuvant. On the 45th day of immunization the animals (*n* = 12) were challenged with 0.2 ml of 5 × LD_100_ doses of crude anthrax toxin (diluted in sterile PBS, pH 7.4) or 0.1 ml of 10 × LD_50_ dose (5 × 10^4^ CFU/ml) of *B. anthracis* Ames spores in PBS, respectively via i.p. route. Mice were observed for morbidity and mortality for 15 days post challenge. The above experiment was performed in containment facility (BSL3) at Department of Biotechnology, Jawaharlal Nehru University, Delhi. The protective efficacy of r-PAbxpB sub unit vaccine against *B. anthracis* toxin and spores was calculated by Kaplan–Meier's method to compare percentage survival.

### Statistical Analysis

The data were represented as mean ± S.D. Mantel-Cox (log rank) test was used to compare the survival curves and Student's *t*-test was used for other statistical comparisons. All graphical illustrations were constructed using Graph Pad Prism 5 software. Significance (P) value summary: ^*^P ≤ 0.05, ^**^P ≤ 0.001; ^***^P ≤ 0.0001.

## Results

### Construction, Cloning, Expression, and Purification of r-PAbxpB

**Figure 1:**
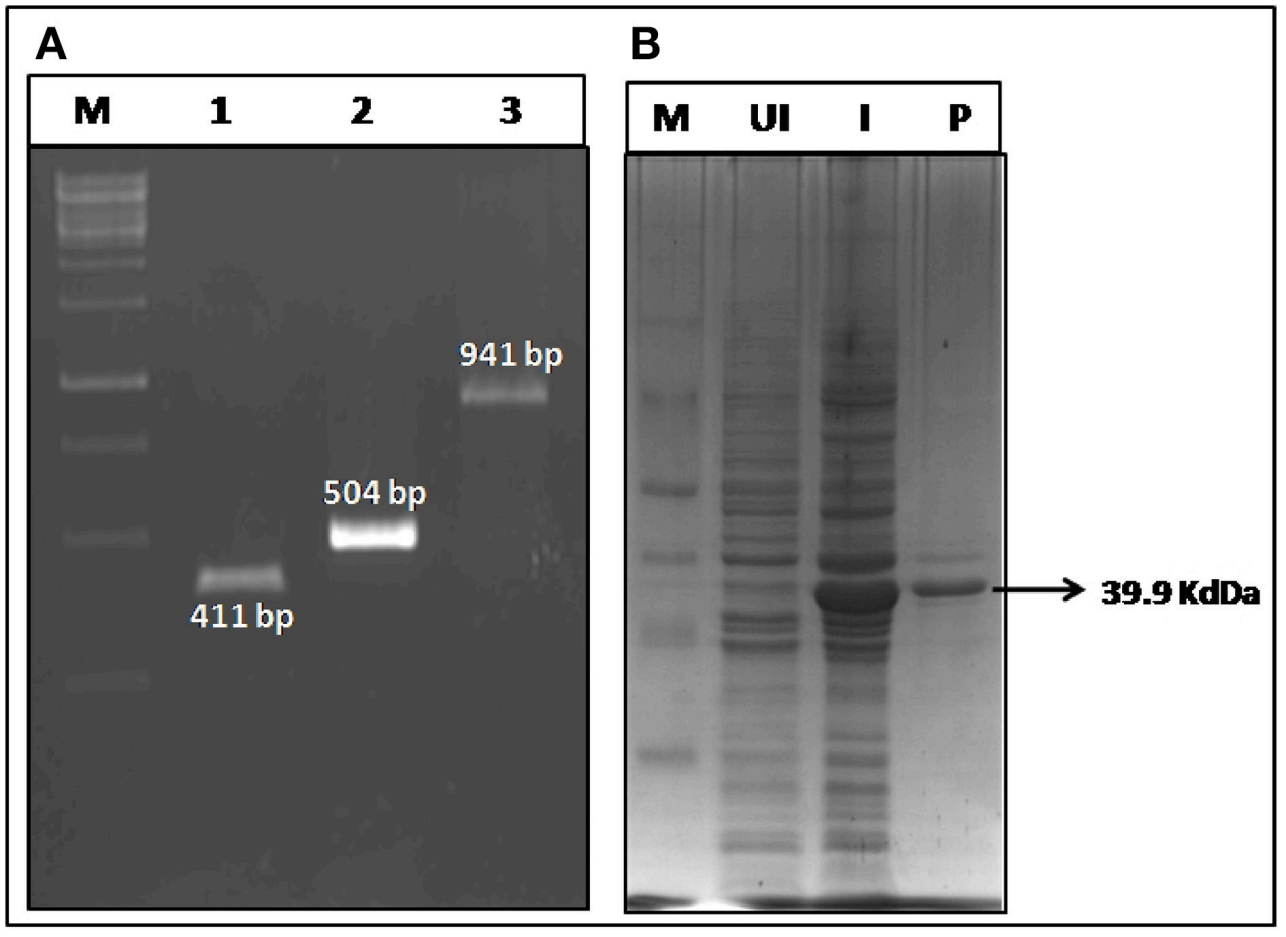
Construction and expression of r-PAbxpB. **(A)** Agarose gel electrophoresis of *pag* (Domain IV) (Lane 1); *bxpB* (Lane 2); *PAbxpB* (Lane 3) amplicons. **(B)** SDS-PAGE (12%) of whole cell lysates from r-PAbxpB *E. coli* clones stained with Coomassie Blue:M-Marker, UI, Un-induced clone; I, Induced Clone; P, r-PAbxpB purified protein.

The bivalent chimeric gene r-*PAbxpB* (941 bp) was constructed by splicing domain IV of PA (*pag* 411 bp) and *bxpB* (504 bp) through a glycine linker (G4S 15 bp) at their 3′ and 5′ ends, respectively by SOE-PCR (Supplementary File 1). The gene was ligated into pRSET A vector by restriction ligation mediated cloning and transformed into *E. coli* BL21 (DE3) and the integrity of cloned gene in r-pRSETA-*PAbxpB* plasmid was authenticated by sequencing. The expression of the r-PAbxpB fusion protein (39.9 kDa) was confirmed in 12% SDS PAGE gel stained with Coomasie Blue (Figure 1). The protein was found to be concentrated in the inclusion bodies and the purification was carried out in denaturing condition using immobilized metal affinity chromatography. A total 20 mg of purified protein was refolded by arginine-PBS mediated dialysis at 4°C. The LPS content in the protein r-PAbxpB protein preparation was below 0.6 EU/ml as determined the Limulus Amoebocyte Lysate (LAL) assay kit (Lonza, India) and was within the prescribed limit (<20 EU/ml) against recombinant proteins.

### Humoral Immune Response of Mice Immunized With r-PAbxpB

**Figure 2:**
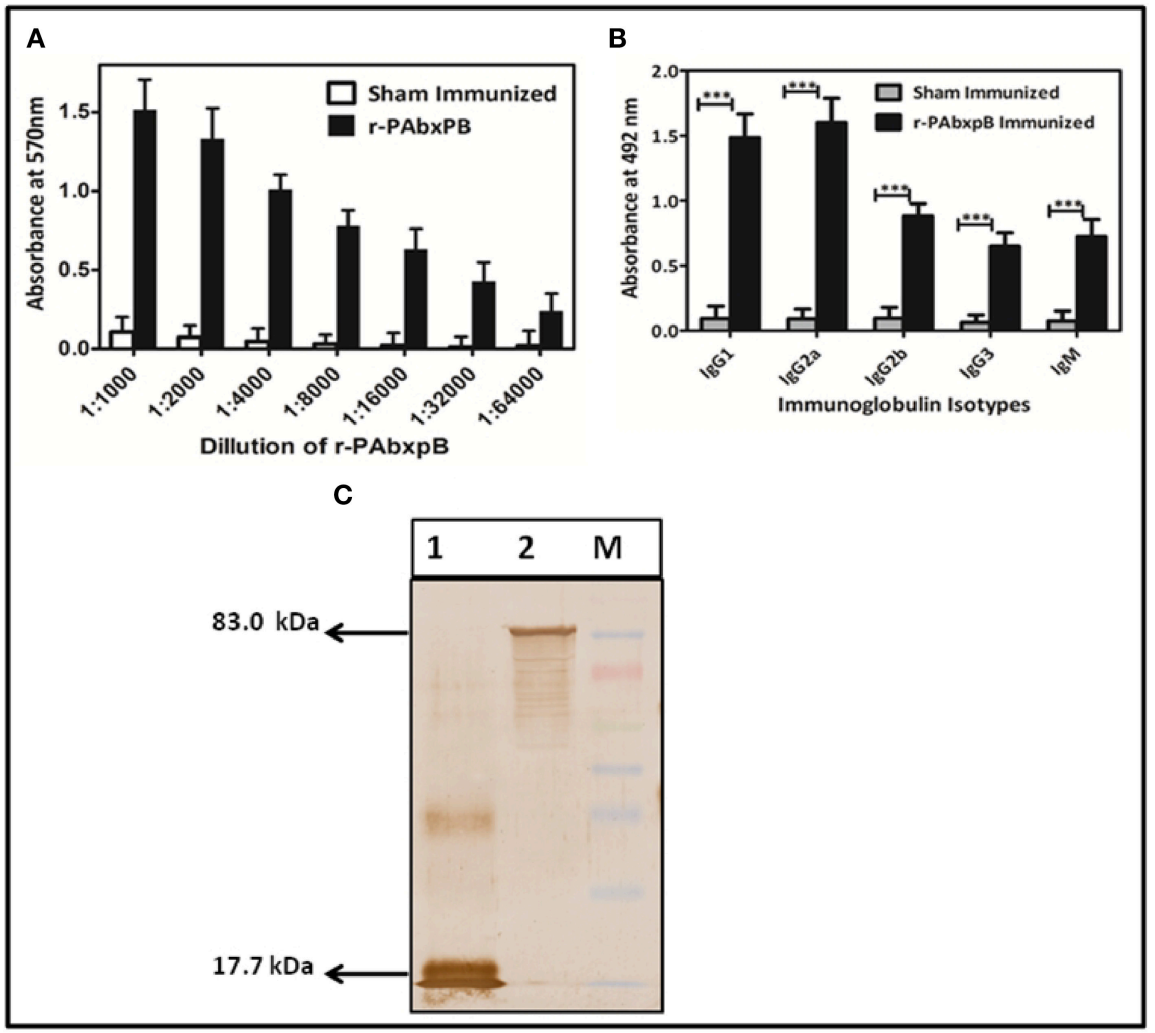
Characterization of r-PAbxpB antibody. **(A)** The end point anti r-PAbxpB titer in sera collected on 0^th^, 14^th^, 28^th^, and 42^nd^ days from 6 randomly selected r-PAbxpB immunized mice as determined by indirect ELISA. **(B)** Determination of r-PAbxpB specific IgG immunoglobulin subclasses on 42^nd^ day (1:1,000^th^ dilution) sera. The experiment **(B)** were performed in triplicates and the data is represented in mean ± S.D. **(C)** Western blot analysis to show the reactivity of anti-r-PAbxpB sera. BxpB (17.7 kDa) (Lane 1) from spore extracts and PA (83 kDa) from *B. anthracis* culture and was found to be statistically significant (p^***^) supernatant (Lane 2). ^***^P ≤ 0.0001.

After the 42^nd^ day of immunization, the group of mice immunized subcutaneously (s.c) with r-PAbxpB exhibited significant and progressive induction of anti-r-PAbxpB antibody titer of 1:64,000 (antilog 4.80618 ± 0.27906) (Figure 2A). The IgG antibody subclasses and IgM levels specific to the fusion protein was analyzed from the sera obtained after the third booster (43^rd^ day). IgG sub typing revealed similar increase of IgG1a and IgG2a antibodies indicating a balanced Th1 and Th2 immune response along with significant amounts of IgM antibodies reflecting the elicitation of innate immune response (Figure 2B). Further the hyper immune sera against anti-r-PAbxpB reacted specifically with PA from *B. anthracis* culture supernatant and BxpB from *B. anthracis* spores generating lucid bands at 83 and 17.7 kDa, respectively (Figure 2C).

### Inhibition of Spore Germination by Anti-r-PAbxpB Antibodies

**Figure 3:**
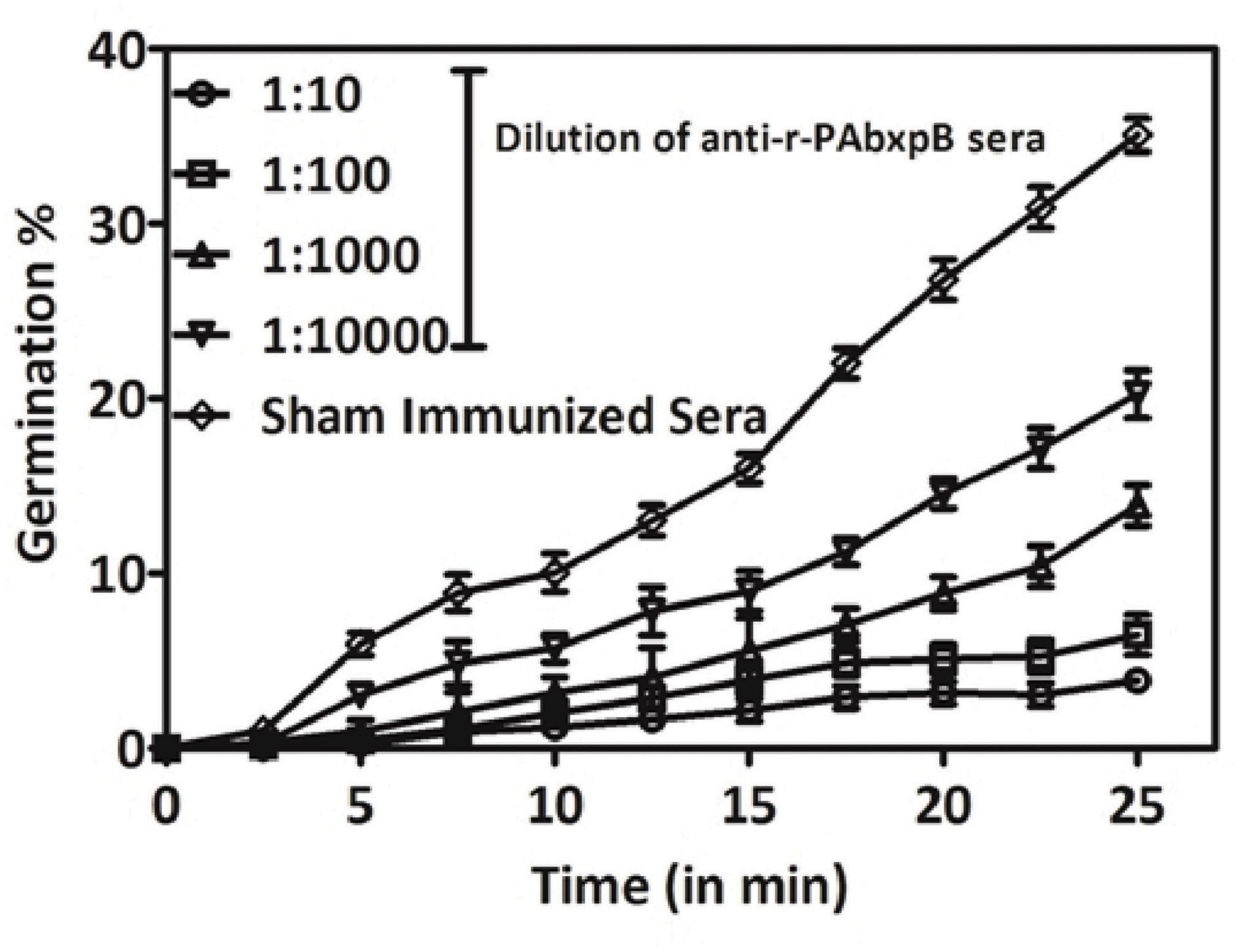
Percentage change in OD_560_ of *B. anthracis* BA10 spores treated with dilutions of anti-r-PAbxpB antisera and incubated in germination media at intervals of 2.5 min till 25 min of incubation. The mean values of data obtained with sera dilution are represented against the sham immunized sera.

The spore germination inhibition capacity of anti-r-PAbxpB antibodies was evaluated on *B. anthracis* spores exposed to different dilutions of antibodies. The germination inhibition capability of the anti-r-PAbxpB antibodies was directly proportional to the concentration of the antibodies. A 1:10 dilution of anti-r-PAbxpB sera resulted in 3.3% spore germination whereas a 1:10,000 dilution resulted in 21.25% germination of spores. In contrast, incubation of spores with sham sera resulted in 38% germination of spores upon 25 min incubation (Figure 3).

### Opsonophagocytosis and Spore Killing by Macrophages

**Figure 4:**
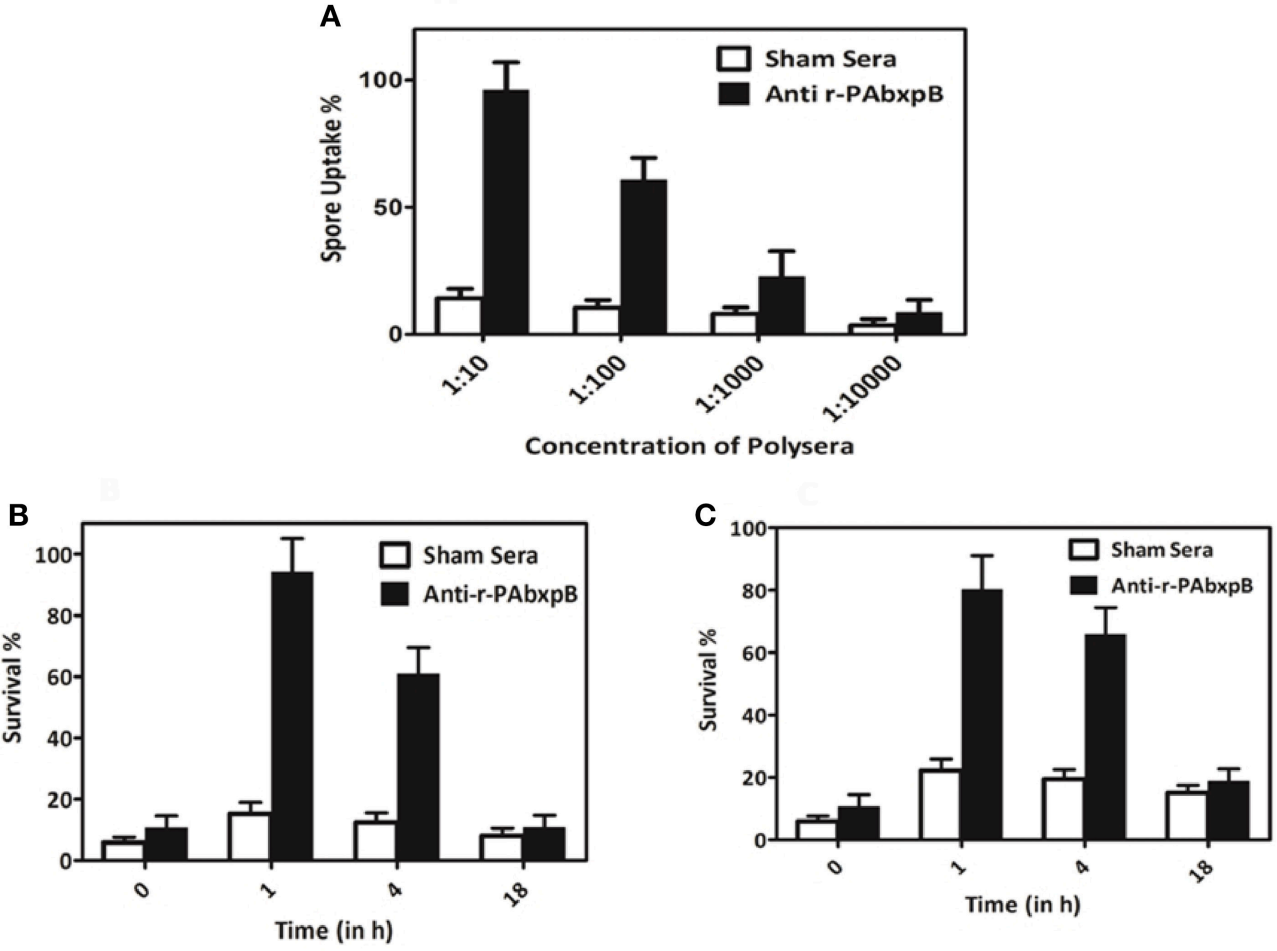
Opsonophagocytosis and killing of spores. Enhanced opsonophagocytosis of *B. anthracis* BA10 spores by macrophage cell lines (Raw 264.7) and *in vitro* killing of spores within the macrophage. **(A)** Uptake of spores pre-treated with 10-fold serial dilutions of anti-r-PAbxpB antibodies or sham sera by Raw 264.7. Macrophage cells after 45 min incubation. Intracellular survival of spores pre-incubated with anti-r-PAbxpB antibodies **(B)** 1:10 dilution and **(C)** 1:100 dilution over time anti-r-PAbxpB antibodies resulted in increased killing of the spores within the macrophages after 4 h of incubation which increased exponentially with time till 18 h. Pre-incubation of spores with sham sera does not result in a significant killing after 4 h and 18 h of incubation. The data in **(A–C)** were obtained from three independent experiments, each performed in triplicates, and the data is represented in mean ± S.D.

The effect of anti-r-PAbxpB antibodies on the opsonization and the survival of *B. anthracis* spores were evaluated on the RAW 264.7 macrophage cell line. A 1:10 dilution of r-PAbxpB immunized sera showed a maximum uptake of 96% while no significant uptake of spores was observed in case of the macrophage cells incubated with sham sera (Figure 4A). The percentage killing of phagocytosed spores is depicted by the number of live phagocytosed spores obtained upon lysing of macrophage cells infected with r-PAbxpB sera treated *B. anthracis* spores. Treatment with 1: 10 dilution of r-PAbxpB immune sera for 18 h resulted in 10% survival of *B. anthracis* spores while 20% spore survival was observed in case of treatment with 1: 100 dilution of r-PAbxpB immune sera post 18 h incubation, respectively (Figures 4B,C). The later depicts that 1: 10 dilution of r-PAbxpB immune sera killed 90% spores within the macrophage cells while 1: 100 dilution killed 80% spores. In contrast the sham sera incubated spores showed no significant killing of spores after similar incubation period.

### *In vitro* Proliferation of Splenocytes From r-PAbxpB Immunized Mice

**Figure 5:**
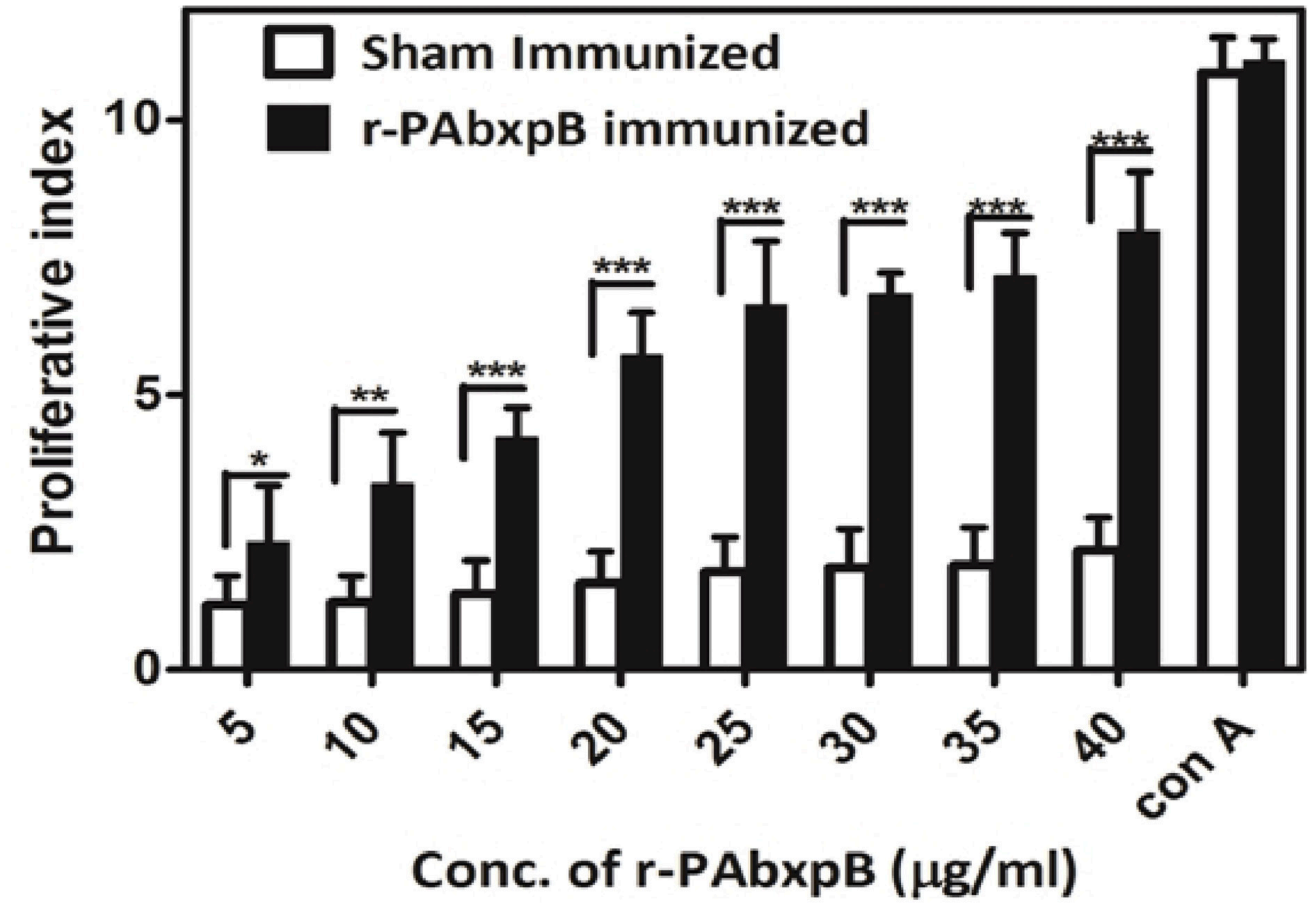
Lymphocyte proliferation assay. Proliferation of r-PAbxpB and sham immunized mice splenocytes re-stimulated with gradient concentrations of r-PAbxpB determined by MTT assay. Conconavalin A at 20 μg was used as positive control. Experiments were performed in triplicates and data represented as mean PI values ± S.D. ^*^P ≤ 0.05, ^**^P ≤ 0.001; ^***^P ≤ 0.0001.

The proliferation of r-PAbxpB and sham immune mice splenocytes upon re-stimulation with r-PAbxpB was evaluated via *in vitro* assays. A significant proliferation (p^***^) PI = 6.43 was observed in the splenocytes from r-PAbxpB immunized mice whereas the sham immunized mice showed no significant increase in proliferative index (PI) when exposed to various concentration of r-PAbxpB antigen. As a positive control, 2 μg/ml of Conconavalin A was used and a significant increase in proliferation was observed in the splenocytes (Figure 5).

### Cytokine Estimation

**Figure 6:**
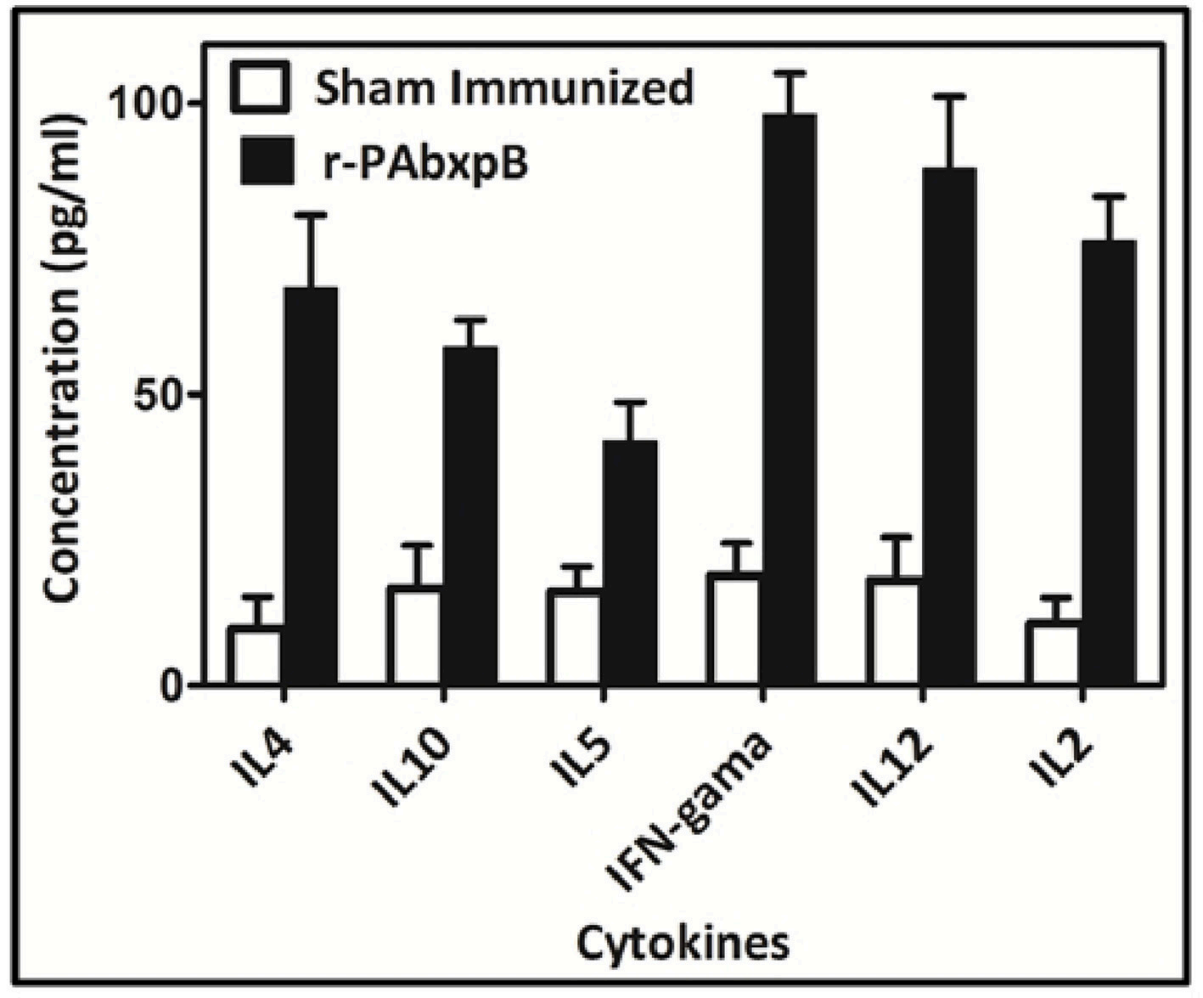
Cytokine profiling in mice immunized with recombinant protein r-PAbxpB. Concentration of Th1 (IFN-γ, IL-2, IL-12) and Th2 (IL-4, IL-5, L10) cytokines in cell culture supernatants of r-PAbxpB, and sham immunized mice splenocytes exposed with r-PAbxpB (30 μg/ml). The experiment was performed in triplicate and the data represent the mean ± SD of the results determined.

The high proliferative index observed in the splenocytes of r-PAbxpB immunized mice prompted us to assess the levels of Th1 and Th2 mediated cytokines expressed in the splenocytes. Splenocytes from mice immunized with r-PAbxpB was exposed to r-PAbxpB (30 μg/ml) and the production of the pro-inflammatory IFN-γ, IL-2, IL-12 anti-inflammatory cytokines (IL-4, IL-5, and IL-10) were evaluated from the culture supernatant of the primed splenocytes. Significantly higher levels of both pro and anti-inflammatory cytokines IFN-γ (5-fold), IL-2 (7-fold), IL-12 (5-fold); IL-4 (7-fold), and IL-5 (3-fold), and IL-10 (3-fold) were observed when compared to the sham immunized splenocytes. As an indirect measure to determine the Th1/Th2 bias IFN-γ: IL-4 ratio was calculated. Since the ratio was 1.2 it was deduced that r-PAbxpB immunized mice indicates a balanced Th1:Th2 response. In contrast the sham immunized mice splenocytes displayed no significant Th1 and Th2 cytokines (Figure 6).

### Flow Cytometric Analysis of CD4+ and CD8+ T-cell Immune Response

**Figure 7:**
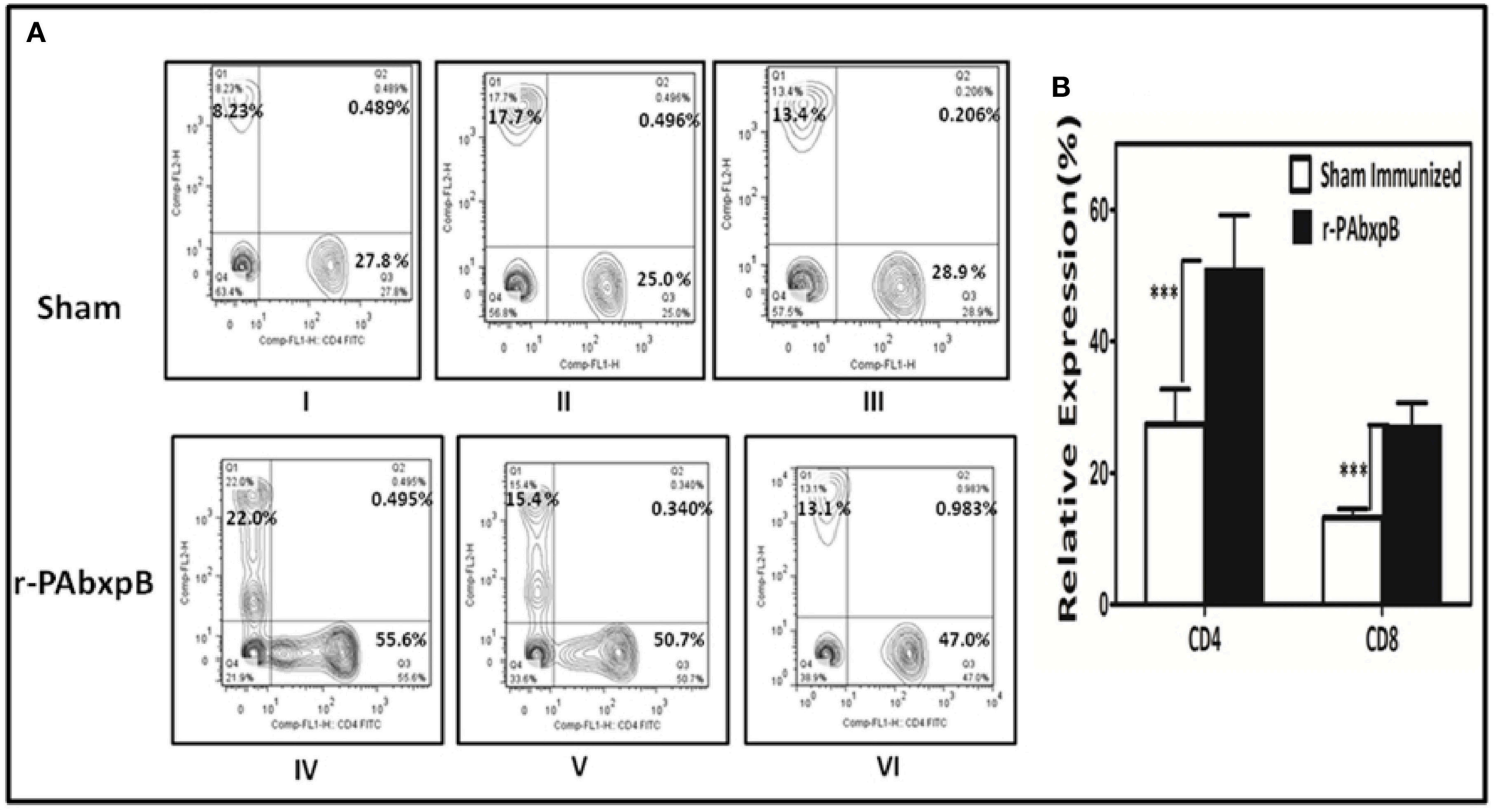
Relative expression of CD4+ and CD8+ T-cells estimated by flow cytometric analysis using FITC anti-mouse CD4 and PE anti-mouse CD8 monoclonal antibodies. Anti-coagulant treated peripheral blood samples from r-PAbxpB and sham immunized mice after 42^nd^ day of immunization were used in this assay. **(A)** Representative graphs showing percentage of CD4+ and CD8+ T cells gated from the T cell population. CD8+ T cell % (Q1), CD4+ and CD8+ T cell % (Q2), CD4+ T cell % (Q3), cell debris (Q4). **(B)** The graphical representation of relative expression percentage of CD4+ and CD8+ cells in r-PAbxpB and sham immunized mice. ^***^P ≤ 0.0001.

The total CD4+ and CD8+ T-cell counts in peripheral blood samples of r-PABxpB immunized and sham immunized mice were evaluated by flow cytometry analysis. Immunization with r-PAbxpB resulted in significantly higher frequency of CD4+ and CD8+ T-cell counts as compared to the sham immune mice with a relatively higher level of CD4+ T cells (51.4%) in comparison to the CD8+ T cells (27.23%) (Figure 7).

### Passive Transfer Studies

**Figure 8:**
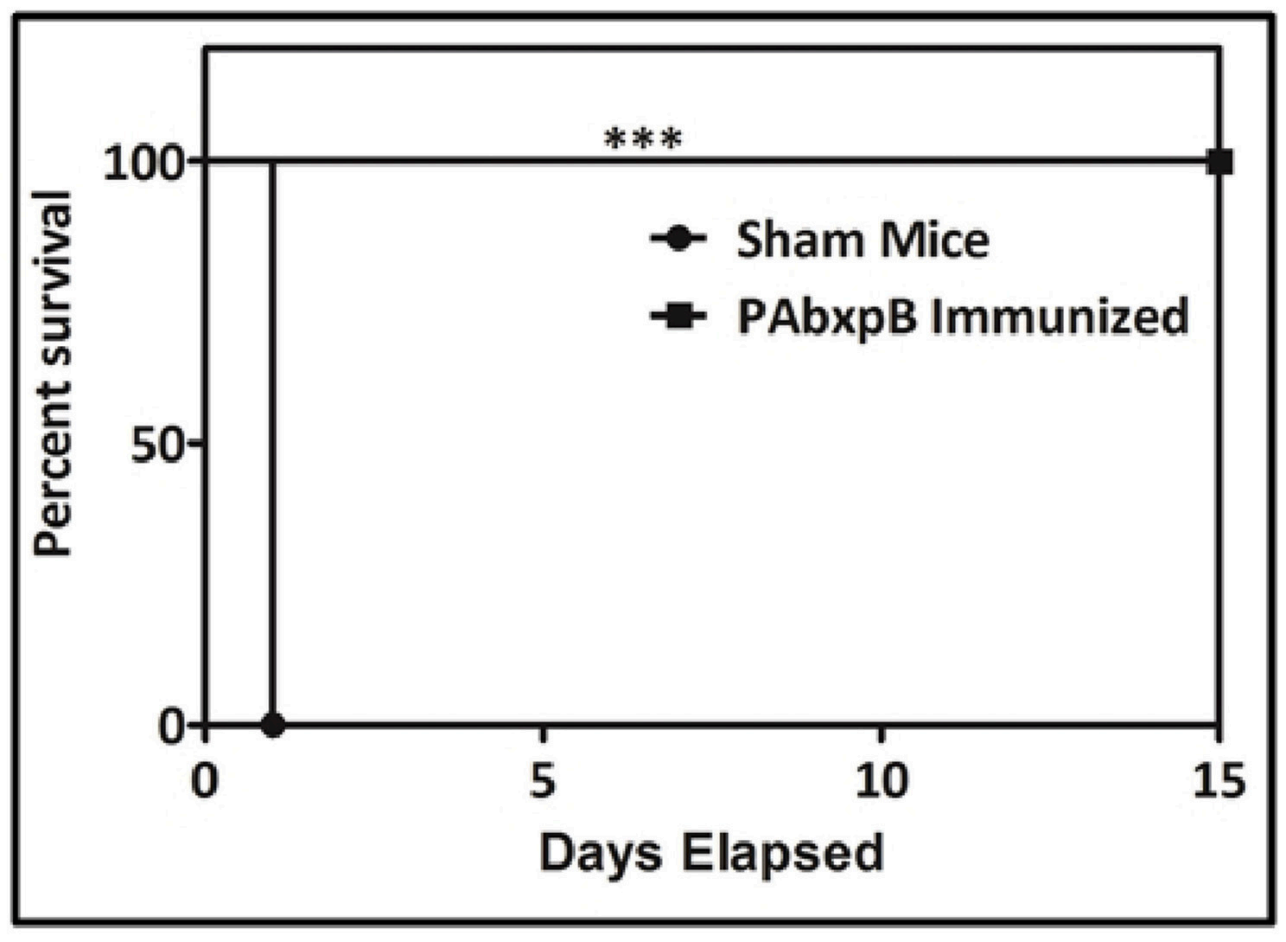
Passive protection of mice against *B. anthracis* toxin challenge. Survival percentages of naive female BALB/c mice (*n* = 6 each group) injected i.p. with anti-r-PAbxpB followed by anthrax toxin after 24 h. The animals were kept for 15 days observation period. Control groups of mice received similar mixtures in polysera from sham immunized mice. Survival percentages were calculated by Kaplan–Meier's method and was statistically significant (p^***^).

Passive immunization of hyperimmune antisera generated against anti-r-PAbxpB protected experimental mice from *B. anthracis* toxemia. The control group which received serum from sham immunized mice before toxin challenge (5 × LD_100_ doses) succumbed to death within 24 h of challenge whereas the anti-r-PAbxpB sera significantly suppressed the toxin induced mortality in the experimental mice resulting in 100% survival (Figure 8). The difference in survival percentage between r-PAbxpB and sham mice was statistically significant (p^***^). These observations confirmed that anti-r-PAbxpB sera can/could effectively neutralize the crude anthrax toxins *in vivo*.

### Active Protection Against *B. anthracis* Spores and Toxin

**Figure 9:**
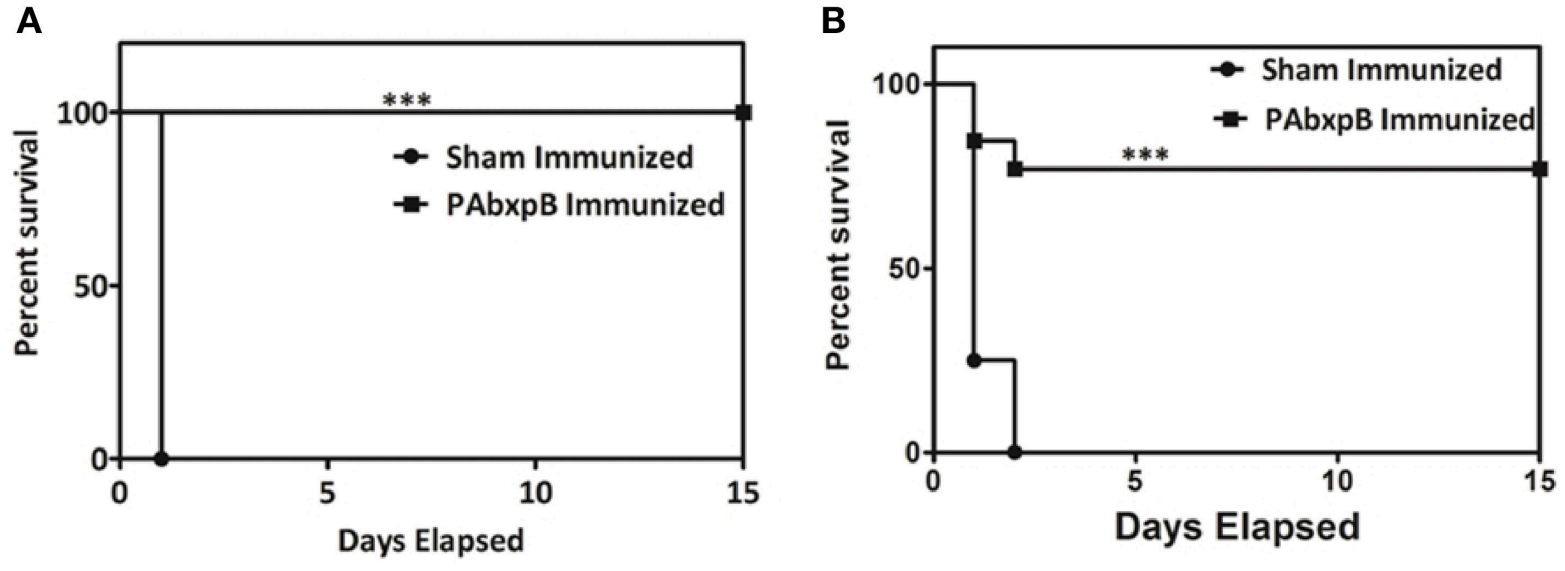
Percentage survival of mice challenged with crude anthrax toxin and spores. Groups of r-PAbxpB, and sham immunized female BALB/c mice (*n* = 12 each group) were challenged i.p. with 5 × LD_100_ units of crude anthrax toxin **(A)** and10 × LD_50_ spores **(B)** on the 45^th^ day of the immunization schedule and survival was monitored for 15 days. The data in **(A,B)** were generated from three independent experiments. The protective efficacy of r-PAbxpB subunit vaccine was calculated by Kaplan–Meier graph to compare percentage survival and was found to be statistically significant (p^***^).

Our next objective was to determine the protective efficacy of r-PAbxpB immunized mice against crude anthrax toxin (5 × LD_100_) or *B. anthracis* Ames strain spores (10 × LD_50_, 5 × 10^4^ CFU/ml). All the r-PAbxpB immunized mice (12/12) survived the toxin challenge whereas 83.3% r-PAbxpB (10/12) immunized mice survived the spore challenge (Figures 9A,B). The sham immunized mice in the control group succumbed to infection with a mean time to death of 24 h after toxin challenge (Figure 9A) and 48 h after spore challenge (Figure 9B). Extensive tissue damage and necrosis of liver, spleen, and intestine was observed in the PBS immunized mice whereas no changes in pathology were observed in the r-PAbxpB immunized mice (data not shown).

## Discussion

Considering the high lethality and poor prognosis associated with anthrax, prophylactic immunization prior to exposure provides protection to potentially vulnerable population (17, 28). The protective antigen (PA) component of anthrax toxins is the major immunogen of currently licensed and next generation anthrax vaccines and is constrained by its instability and inability to restrict bacterial proliferation within the host (13). Alternatively, immunizations with live non-virulent or formaldehyde inactivated spores (FIS) or recombinant spore components generates anti-spore immune responses and augments anti-anthrax protective immunity when combined with PA (13, 29, 30). BxpB localized in the exosporium basal layer plays a crucial role in exosporium organization and is reported to augment PA protective efficacy by improved phagocytic spore clearance when administered as a booster following r-PA83 immunization (17). The domain IV (PAIV) of PA maintains structural integrity even at low pH conditions and binds to host cell receptors while generating anti anthrax protective immunity similar to entire PA (31, 32). Therefore, in the present study PAIV was spliced with BxpB via a flexible G4S linker to generate a single functional antigen r-PAbxpB to elicit protective immune response that prevents establishment of infection by the spores and also neutralize anthrax toxins. The addition of G4S linker surmounted the problem of stearic hindrances and antigenic competition otherwise observed during administration of fusion proteins without linkers or antigen cocktails, respectively (17, 33).

The r-PAbxpB bivalent protein was found to be immunogenic eliciting high titers of circulating antibodies (1:64,000) (Figure 2A) that reacted lucidly (Figure 2C) with both PA (83 kDa) and BxpB (17.7 kDa) from *B. anthracis* toxin and spore extracts, respectively in Western blot. The reactivity of the antibodies confirms that integrity of epitopes from both PAIV and BxpB were maintained in the fusion protein and antibodies were produced against both the components (Figure 2C). Antibodies binding to the BxpB act as opsonins and help host immune system to restrict the spores from establishing infection by enhancing their uptake and killing within the macrophages (17). BxpB when administered as a cocktail along with r-PA (PA83) could confer 70% spore killing within the macrophage (17). In the present study, the anti-r-PAbxpB antibodies effectively enhanced spore phagocytosis (96% uptake Figure 4A) and resulted in 90% spore killing (Figures 4B,C) by the macrophages. The later can possibly be attributed to high titer opsonizing anti r-PabxpB antibodies with significant levels of IgM subclass elicited by the cumulative effect of administering PA IV and BxpB antigens as a fusion protein; use of Freunds adjuvant and multiple boosters (34). Further the anti-r-PAbxpB treated spores exhibited 3.3% germination (Figure 3) when compared with those exposed to sham sera (38%). This spore germination inhibition could possibly be attributed to the anti PAIV component of anti r-PAbxpB antibodies as it is reported that anti-PA antibodies retard spore germination while anti-BxpB antibodies do not possess spore germination inhibition properties (17).

The *B. anthracis* vegetative cells after multiplication in the host system release anthrax toxin factors that get internalized into host cytosol through PA mediated binding to ANTXR1 and ANTXR2 receptors (35). The anti-PA antibodies play an effective intervention mechanism in anthrax pathogenesis (36). In the present study, passive transfer of the anti-r-PAbxpB antibodies resulted in 100% survival against the anthrax toxin challenge while all animals in control group immunized with sham immune sera died due to severe toxemia (Figure 3). The anti-PAIV component of the anti-r-PAbxpB antibodies possibly obscured the binding region of PA83 preventing subsequent heptamerization and the release of LT and ET into the cytosol. Hence the tissue necrosis in liver, spleen and intestine, and cell death (37) otherwise observed during the infection process was not observed in our *in vivo* assays

Active immunization with r-PAbxpB generated CD4+ T-helper cell response that in turn elicited induction of IL-2, IL-12, and IFN-γ (Th1) and IL-4, IL-5, and IL-10 (Th2) cytokines (Figure 6). The precursor cytokine IL-12 induces the production of principle Th1 effector cytokine IFN-γ. The later plays an important role in restricting initial anthrax infection by inducing antibodies with spore opsonization and phagocytosis properties and activating the nitric oxide mediated spore killing by the macrophages (34, 38). Therefore, the r-PAbxpB mediated increase in IL-12 and IFN– γ production along with an upsurge in the complement fixing and opsonizing antibody subclass IgG2A (Figure 2B) can be correlated with the opsonophagocytosis (Figure 4A) and macrophage killing reported in the present study (Figures 4B,C). Though, predominantly an extra cellular pathogen, the residence of phagocytosed *B. anthracis* spores within macrophage denotes the intra cellular phase wherein the spores get transported to the lymphatics for systemic dissemination. T cell mediated eradication of intracellular pathogens involves IL-2 mediated proliferation of cytotoxic CD8 T cells that help in killing the intracellular bacteria by lysing the infected cells (39). We therefore speculate that the 7-fold up regulation of IL-2 and 2-fold increase in CD8 cells in our study could be contributing to protection by purging/lysing spore bearing macrophage cells. Alternatively, the r-PAbxpB mediated induction of Th2 cytokines (IL-4, IL-5, and IL-10) could be correlated with enhanced B cell survival and antibody production as evidenced by high titers of anti-r-PAbxpB specific antibodies with dominance of non-complement fixing antibody isotype IgG1 (Figure 2A). Further, the induction of IgG1/G2a and IFN-γ/IL-4 in 1:1 ratio indicated the anti-inflammatory bias otherwise observed for IL-10 was insignificant in the present study.

Active immunization of mice with r-PAbxpB resulted in 83.3% survival against Ames spore challenge and 100% protection against toxin challenge whereas, in our earlier study, rPA immunization resulted in 100% protection against toxin challenge and 50% protection against spore challenge (22) confirming the superior protective efficacy of r-PAbxpB over r-PA. The protective immunity provided by r-PAbxpB could be attributed to the following activities. The effective antispore immune response viz, germination inhibition, enhanced spore phagocytosis, and spore killing by the macrophages, elicited by the r-PAbxpB molecule possibly interfered with the proliferation and systemic spread events associated with the early phase of anthrax infection, while the toxin neutralizing antibodies hindered the immunomodulatory and cytotoxicity effect of anthrax toxins in the later phase. The present study thus describes r-PAbxpB as an efficient vaccine candidate with the capability to induce enhanced CD4+ T cell proliferation along with synergistic upregulation of both Th1 and Th2 subsets and CD8+ T cell proliferation. The protective role played by r-PAbxpB primed cytotoxic CD8+ T cells in purging infected macrophages or other host cells and effect of adjuvants to improve protective efficacy needs to be established by further studies.

## Author Contributions

Experiments carried out in this study were performed by SM, SD, and SSM. SM, SD, and JK were involved in designing of experiments, data interpretation, and manuscript preparation. RB and VS were involved in challenge studies data interpretation and manuscript preparation. All authors read and approved the final manuscript.

### Conflict of Interest Statement

The authors declare that the research was conducted in the absence of any commercial or financial relationships that could be construed as a potential conflict of interest.
