# Supplementary material for: A Bivalent Protein r-PAbxpB Comprising PA Domain IV and Exosporium Protein BxpB Confers Protection Against B. anthracis Spores and Toxin
Source: Front Immunol. 2019 Mar 19;10:498. doi: 10.3389/fimmu.2019.00498 (PMC6433990; doi:10.3389/fimmu.2019.00498)
Supplement: Supplementary file 1 [file Data_Sheet_1.PDF]

A bivalent protein r-PAbxpB comprising PA domain IV and exosporium protein BxpB confers protection against *B. anthracis* spores and toxin

Saugata Majumder[1], Shreya Das[1], Vikas Somani[2], Shivakiran S. Makam[1], Joseph Kingston J[1]*, Rakesh Bhatnagar[2]

*Corresponding Author

[1]Microbiology Division, Defence Food Research Laboratory, Defence Research Development Organisation, Mysore-570011

[2]School of Biotechnology, Jawaharlal Nehru University, New Delhi 110067

Corresponding Author details:

Email: joseph@dfrl.drdo.in

Ph: +91-821-2579435

Materials and Methods

Construction of *PAbxpB* Chimeric Gene by splicing overlap extension PCR:

DNA of *B.anthracis* BA10 was isolated following Marmur (1961). Nucleotide sequences encoding for PAIV (1930-2340 bp) of *pag* gene (Genbank accession AF306782.1) and the entire *bxpB* gene of 504 bp (Genbank accession NC_005945.1) were spliced by overlap-extension PCR via the nucleotide fragment (GGTGGTGGTGGTTCA) encoding a flexible glycine linker (15 bp) to form a chimeric gene (*PAbxpB*).

The whole strategy comprised of three important steps.

Step1. Amplification of individual fragments with complementary overhangs

Gene fragments encoding domain IV of *pag* (411 bp) and *bxpB* (504 bp) were PCR amplified independently by PAF + PAR and bxpBF + bxpBR primers respectively. In another PCR, glycine linker overhangs were added to modify both the *pag* (PAF+ PAGlyR primer sets) and *bxpB* (bxpBF+ bxpBGlyR primer sets), at 3' and 5' end respectively.

Step2. Single Step Fusion PCR

Equimolar ratios of the modified gene fragments were mixed with each other and were spliced together by primer free PCR.

Step3. Nested Amplification of fused gene.

The spliced products were amplified using the extreme primer PA-CLN-F and bxpB-CLN-R to include the restriction sites *Xho*I and *Hind* III at the 5' and 3' ends of the chimeric gene respectively.

PCR Conditions:

Except the PCR in Step 2, all other PCRs (Mastercycler Pro, Eppendorf, Germany) were kept in 20 µl reaction mix containing 50 ng template DNA, 1X *Pfu* buffer (with 2.5 mmol^{-1} $MgSO_4$), 0.2 mmol^{-1} dNTP mix, 10 pmol^{-1} each primer and 1 unit *Pfu* polymerase (Fermentas, New Delhi, India). The PCR conditions were as follows: initial denaturation at 94 °C for 4 min, 30 cycles of 1 min denaturation at 94 °C, 1 min annealing at 56 °C, 1 min extension at 72 °C and a final extension at 72 °C for 8 min. The fusion PCR was performed in a 30 µl reaction mix containing 100 ng each of purified *pag* and *bxpB* fragments (PCR-Clean up kit; Sigma-Aldrich), 1X *Pfu* PCR buffer (with 2.5 mmol^{-1} MgSO4), 0.2 mmol^{-1} dNTPs mix and 2 unit's *Pfu* polymerase (Fermentas, New Delhi, India). The PCR conditions for OE-PCR were denaturation at 94 °C for 5 min, annealing at 56 °C for 1 min and extension at 72 °C for 15 min. All the amplicons were electrophoresed in 1% agarose gel, stained with ethidium-bromide and visualized under UV Transilluminator.

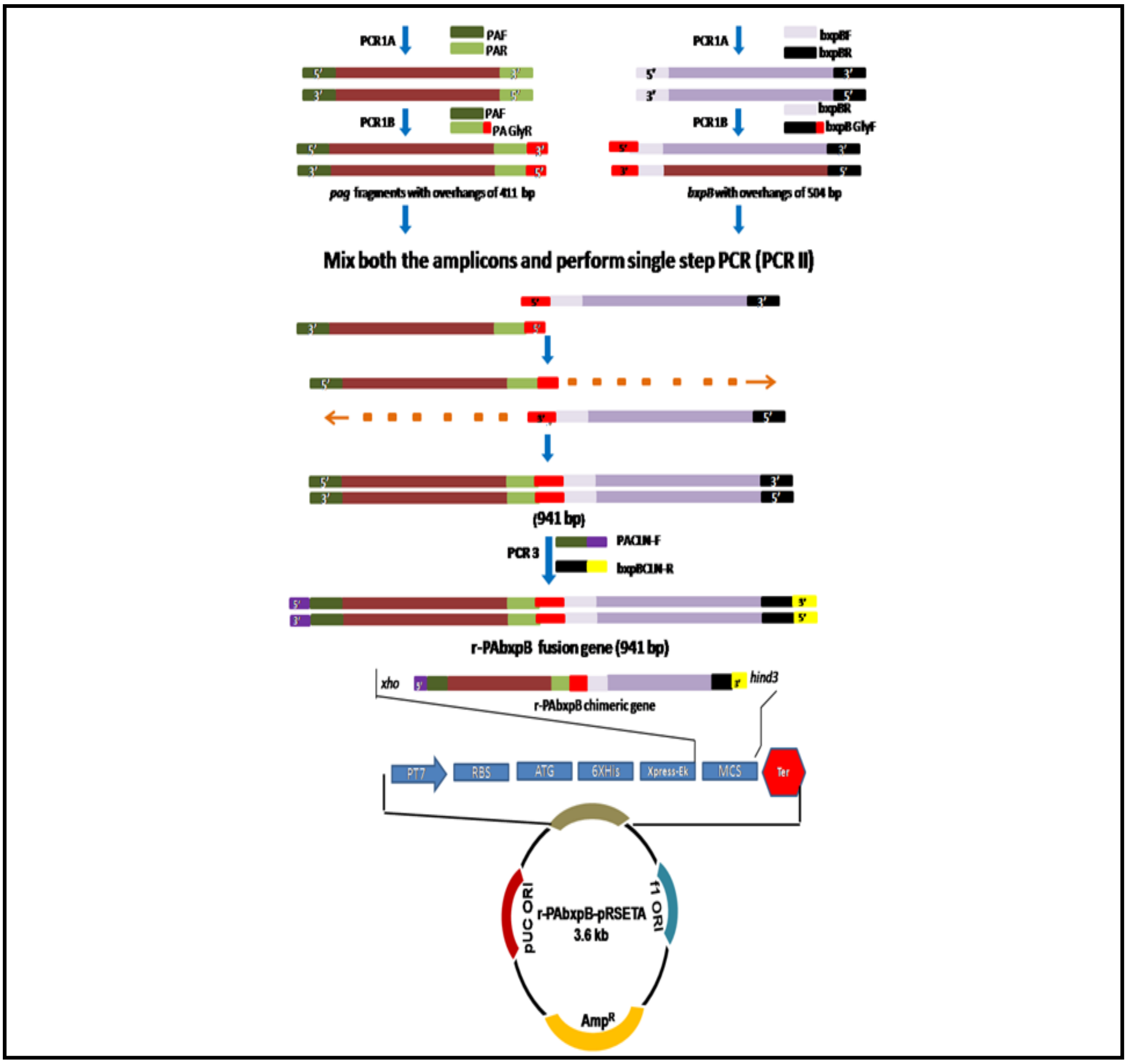

Schematic representation of construction of r-PAbxpB gene by OE-PCR

Cloning, Expression and Purification of PAbxpB chimera

The chimeric gene *PAbxpB* was PCR amplified using the PA-CLN-F and bxpB-CLN-R primers, which added restriction sites for *Xho* I (underlined in forward primer) and *Hind* III (underlined in reverse primer). The PCR product and pRSET A vector (Invitrogen, Bangalore, India) were digested with corresponding restriction enzymes (NEB, Ipswich, UK) and ligated together to form pRSET A- PAbxpB recombinant vector. The recombinant vector was transformed into *E. coli* BL21 DE3 (Invitrogen), and clones were screened by PCR. Overnight grown positive cultures were re-inoculated into fresh LB broth (1:100) and

incubated at 37 °C with shaking until an OD of 0.6 was reached. The culture was induced with 1 mmol^{-1} Isopropyl b-D-thiogalactopyranoside (Sigma, India) for 5 h. Bacteria were harvested by centrifugation at 10,000 g for 10 min, suspended in 1/10th volume PBS (pH 7.4) and analysed for expression by 12 % SDS-PAGE. Expression positive clones were induced in bulk, and the recombinant protein, r-PAbxpB, was purified under denaturing conditions by Immobilized Metal Affinity Chromatography (IMAC) using Ni^{2+}-NTA slurry (Qiagen, Hilden, Germany) according to the manufacturer's protocol. The purified protein was dialysed against PBS + 10 mmol^{-1} arginine (pH 7.4) for 4 h at 4 °C, and the refolded protein was ascertained by SDS-PAGE and quantified by Lowry's method against known bovine serum albumin standards.

Primers for OE-PCR

Primer	Sequence	No of Bases	Size (bp)
PA-F	GATAGAAATAACATAGCAGTTG	22	411
PA-R	TCCTATCTCATAGCCTTTTTT	21	
bxp-F	ATGTTCTCTTCTGATTGCGA	20	504
bxpB-R	CTAGCTAATTTGTGCAACTG	20	
PA-CLN-F	CCGCTCGAGGATAGAAATAACATAGCAG	28	941
PA-GLY-R	GAGAACATTGAACCACCACCACCTCCTATCTCATAGCCTT	40	
bxpBGLY-F	AGGAGGTGGTGGTGGTTCAATGTTCTCTTCTGATTGCGA	39	
bxpBCLN-R	GGGAAGCTTCTAGCTAATTTGTGCAACTG	29	

Reference:

1. Marmur, J. A procedure for the isolation of deoxyribonucleic acid from micro-organisms. *J Mol Biol* (1961) 3(2): 208IN1-218
